# Peroral Endoscopic Myotomy for the Treatment of Achalasia: An Analysis

**DOI:** 10.1155/2013/389596

**Published:** 2013-10-27

**Authors:** Dennis Yang, Mihir S. Wagh

**Affiliations:** Division of Gastroenterology, University of Florida, 1600 SW Archer Road, Room HD 602, Gainesville, FL 32610, USA

## Abstract

Achalasia is a motility disorder of the esophagus, characterized by aperistalsis of the esophageal body and incomplete relaxation of the lower esophageal sphincter (LES). Treatment of achalasia is currently aimed at decreasing the resting pressure in the LES. Peroral endoscopic myotomy (POEM) is an emerging novel endoscopic procedure for the treatment of achalasia with initial data suggesting an acceptable safety profile, excellent short-term symptom resolution, low incidence of postprocedural gastroesophageal reflux (GER), and improvement in manometric outcomes. Further prospective randomized trials are required to evaluate the long-term effectiveness of this promising technique compared to other treatment modalities for achalasia. In this review we outline the technical aspects of POEM, summarize the available data on safety and outcomes, and suggest future directions for further advancement of this minimally invasive approach for the treatment of achalasia.

## 1. Introduction

Achalasia is a motility disorder of the esophagus, characterized by aperistalsis of the esophageal body and incomplete relaxation of the lower esophageal sphincter (LES). In some cases, the underlying pathophysiology has been attributed to the selective loss of inhibitory ganglion cell function in the myenteric plexus [1]. Typical clinical symptoms include dysphagia for solids and liquids, food regurgitation, and retrosternal chest pain. The diagnosis of achalasia is based on clinical, endoscopic, and radiographic findings, with esophageal manometry considered the gold standard [2, 3]. Treatment of achalasia is currently aimed at decreasing the resting pressure in the LES to facilitate the passage of ingested food.

Pharmacological therapy with agents that reduce LES pressure, such as nitrates and calcium antagonists, has yielded unsatisfactory results and has been associated with significant side effects [3]. Endoscopic delivery of botulinum toxin into the LES muscle decreases sphincter tone by inhibiting acetylcholine release from smooth muscle excitatory neurons. Unlike nitrates or calcium antagonists, botulinum toxin injection has been widely used because of its adequate immediate response (success rates of over 90%) and overall safety profile. However, long-term results have been disappointing, with only half of all patients benefiting for more than 1 year [4]. Thus, in clinical practice, this therapy is mainly confined to treating older patients or those with unacceptable surgical risks [4, 5].

Given the relatively low efficacy of pharmacological therapies to date, definitive treatment has focused on the mechanical disruption of the LES. Endoscopic disruption of the LES involves pneumatic dilation of the sphincter with an air-filled balloon. The balloon is inflated, most often under fluoroscopic guidance, until the waist caused by the LES is obliterated. A recent meta-analysis of 29 studies by Katzka and Castell [6], evaluating pneumatic dilation for achalasia with Rigiflex balloons, revealed an 88% 1-year efficacy, gradually decreasing with time to 70 and 29% after 5 and 10 years, respectively. While the results suggested a decline in efficacy and symptom recurrence with time, periodic redilation was associated with sustained response rates of 81–93% after 6–10 years [6, 7]. Alternatively, from a surgical perspective, disruption of the LES can be achieved by myotomy of the smooth muscle sphincter. Several retrospective studies on laparoscopic Heller myotomy (LHM) have reported high rates of symptomatic resolution (80–84%) in patients at a mean follow-up of 5 to 6 years [8, 9]. Congruent with these results, in a meta-analysis including 3086 patients, Campos et al. described success rates with surgical myotomy as high as 89% (range 77–100%) after a mean follow-up of 35 months [10]. Because of these favorable outcomes, LHM is considered the operative gold standard of care by many authorities [11]. However, the disadvantages of LHM are that it is an invasive surgical procedure, necessitates complete mobilization of the gastroesophageal junction, and often requires an adjunctive antireflux operation.

With the exponential advance in natural orifice translumenal endoscopic surgery (NOTES), an interesting novel endoscopic method for the treatment of achalasia was first described by Pasricha and colleagues in a porcine model [12]. The authors demonstrated the feasibility of endoscopic myotomy by directly approaching the esophageal muscular layer through a submucosal tunnel. Subsequently, this technique of peroral endoscopic myotomy (POEM) was translated into clinical practice by Inoue et al. in 2010 [13]. Since then, there have been several prospective studies evaluating the feasibility, efficacy, and safety of this innovative endoscopic technique for the treatment of achalasia [14–25].

## 2. Technical Aspects

Multiple centers have performed POEM as initially described by Inoue et al. in 2010, with some technical variations. We summarize the POEM technique based on the current prospective studies and recent results from the international POEM survey [26].

### 2.1. Preoperative Patient Preparation

Patients selected to undergo POEM should have a diagnosis of achalasia firmly established by different methods (esophageal manometry, esophagogastroduodenoscopy (EGD) and esophagram). Patients are generally maintained on a clear liquid diet for 24–48 hours prior to the procedure. Prophylactic antibiotics are universally initiated on the day of the procedure, continued for the length of postprocedural hospitalization, and, in some cases, extended to 7 days. 

### 2.2. POEM Operative Technique

#### 2.2.1. Submucosal Tunnel Creation

All reported POEM cases have been performed with the patient under general anesthesia. In general, the patient is placed in the supine position and EGD is performed with a forward-viewing upper endoscope and minimal carbon dioxide (CO_2_) insufflation. In most institutions, a dissecting cap is fitted onto the tip of the endoscope to facilitate its insertion into the submucosal space. With 12 o'clock representing the most anterior aspect of the esophagus on endoluminal view, most practitioners favor a mucosal incision site in the 1 to 2 o'clock position on the right anterolateral esophagus, in order to aim for a straight tunnel ending at the lesser curvature of the stomach. However, some centers favor a posterior approach in the 5 o'clock orientation, which is often regarded as the preferred site in patients with previous surgical myotomy, as POEM could be complicated by scar tissue in the anterior plane [20]. The site for mucosotomy in the midesophagus is often selected to be approximately 14 cm from the GEJ. Water is instilled into the esophagus and pooling of saline posteriorly is used to confirm anterior-posterior orientations. An anterior submucosal “lift” is achieved by injecting saline, glycerol-based solutions, or hydroxypropyl methylcellulose + MESNA. A triangle-tip (TT) endoscopic cautery knife (Triangle Tip Knife; Olympus) or a T-type hybrid (HK) knife (ERBE) is used to make a 1.5–3 cm longitudinal mucosal incision at the site of the submucosal bleb. The endoscope is subsequently inserted into the newly created submucosal space. Using a combination of blunt dissection with the cap and electrocautery, a submucosal tunnel is created. CO_2_ insufflation and saline infusion are performed to facilitate submucosal tunneling, while repeated injections of dye help identify the correct plane and mark progression. The blue dye is used as a guide as it stains the muscle layer but not the loose areolar tissue of the submucosa. As the GEJ is approached, the tunnel becomes distinctively narrower. After passing this anatomical landmark, the space in the tunnel widens with a change in the muscle structure from clear circular fibers to irregular fibers, corresponding to the transition to the gastric cardia. Once the submucosal tunnel has been extended caudally, more dye is injected and the endoscope is withdrawn from the tunnel and advanced into the gastric lumen to endoscopically confirm that the distal end of the tunnel is at least 3 cm past the GEJ (i.e., blue dye is seen endoscopically extending at least 3 cm beyond the GEJ).

#### 2.2.2. Submucosal Dissection

The endoscope is reinserted into the submucosal tunnel and dissection of the circular muscle layer is initiated approximately 3-4 cm distal to the mucosal entry site (approximately 10 cm above the GEJ), assuring that the breach of the mucosa and the muscle is not at the same level so as to minimize risk of esophageal leak after POEM. Dissection proceeds from proximal to distal, with an effort to maintain the underlying longitudinal fibers undisturbed. Full myotomy with splitting of the longitudinal fibers is often inevitable but theoretically inconsequential as long as there is no mucosal breach. Coagulation forceps are utilized for hemostasis of submucosal vessels when needed. Upon completion of the myotomy, the endoscope is withdrawn from the submucosal space.

#### 2.2.3. Closure of Mucosal Entry Site

The mucosal entry site is usually closed by endoscopic clips. The initial clip is placed at the most distal part of the incision to facilitate approximation of the incisional borders and placement of subsequent clips is done in a proximal direction to completely close the mucosotomy site. In cases in which the sides of the incision are difficult to approximate, over-the-scope clips have been employed [27].

### 2.3. Intraoperative Monitoring and Care

Inspiratory pressures (plateau pressures) and abdominal distension is monitored continuously during POEM. Elevation of the plateau pressure from baseline and, or abdominal distension is promptly treated by withdrawing the endoscope from the tunnel and advancing it into the gastric lumen to decompress the stomach by suctioning excess CO_2_. Capno/pneumoperitoneum is decompressed by placing a 14 G angiocatheter into the abdomen.

### 2.4. Postoperative Care

Patients are kept nil per os (NPO) the night after the procedure and started on an intravenous proton-pump inhibitor (PPI) immediately postoperatively, which can be subsequently transitioned to oral therapy upon resumption of diet. A gastrografin esophagram is obtained the next day to assess the effect of myotomy and to identify complications such as leaks. There is no consensus on whether a “second look” EGD within 24–72 hours following POEM is indicated, as it often does not affect the management of these patients [26]. Patients are generally discharged within 3 days of postoperative care if no complications are seen clinically and on esophagram. A full liquid diet is recommended for one week after POEM and then gradually advanced.

## 3. Published Data

A literature search in Medline/PubMed was conducted to identify all studies in the English language on POEM for the treatment of achalasia up to May 2013. Keywords searched included peroral endoscopic myotomy, POEM, achalasia, Heller myotomy, pneumatic balloon dilation, and dysphagia. 

### 3.1. POEM for the Treatment of Achalasia

Table 1 summarizes the published clinical data on POEM from various institutions. In the first clinical study of 17 patients, Inoue and colleagues described performing mucosotomy at the level of the midesophagus, allowing submucosal insertion of the endoscope [13]. Complete division of the inner circular muscle layer was achieved by combining blunt dissection and electrocautery and this was continued for a few centimeters distal to the GEJ in the proximal stomach. Upon completion of the myotomy, the mucosal entry site was closed with endoclips. The investigators showed that endoscopic myotomy was technically feasible by completing the procedure in all of the patients. 

Pneumoperitoneum occurred in one patient, who was treated successfully by puncturing the abdominal wall with an 18-gauge needle. None of the 17 patients had postoperative subcutaneous emphysema on clinical grounds nor reported complications or problems during the follow-up period. The authors reported a significant reduction in the dysphagia symptom score (10 to 1.3; *P* = 0.0003) and the resting LES pressure from 52.4 (14.2–80.5) to 19.8 (9.3–42.7) (*P* = 0.0001) after POEM during short-term follow-up (mean 5 months). 

There have been several studies since then, which have confirmed the high success rate with this technique as initially reported in the landmark study by Inoue and colleagues. In 2011, Swanström et al. reported their experience with POEM in 5 patients [14]. Technical success, based on completion of the procedure, was achieved in all 5 patients. At the 2-week clinical follow-up, all patients reported immediate symptom relief. Similarly, other centers have independently reported excellent short-term symptom resolution rates with POEM [15–20]. Using the Eckardt score, a validated symptom score for achalasia, the majority of patients (97%; range 91–100%) achieved an Eckardt score ≤3 after POEM at 3-month follow-up. Symptomatic improvement after POEM was also accompanied by a decrease in mean LES pressure on follow-up manometry (Table 1). In addition to favorable Eckardt scores and manometric outcomes, Chiu et al. [17] also suggested that POEM improved esophagogastric function, based on esophageal emptying on timed barium esophagram and esophagogastric junction distensibility on EndoFLIP (Crospon, Galway, Ireland).

In light of all these studies supporting the feasibility and safety profile of POEM, Hungness and colleagues published the results from their clinical trial comparing POEM and LHM [21]. In this study, 18 patients underwent POEM and were compared to a control group of 55 LHM patients enrolled in a prospective outcomes database. The authors demonstrated slightly shorter operative times for POEM compared to LHM (113 versus 125 minutes, *P* < 0.001) and less estimated blood loss (≤10 mL in all cases versus 50 (10–250) mL, *P* < 0.001). Surprisingly, pain scores were similar upon postanesthesia care unit arrival and postoperative day 1 but were higher at 2 hours for POEM patients (3.5 versus 2, *P* < 0.03). The authors reported no differences in minor complications and there were no mortalities in either group. In terms of treatment efficacy, high-resolution manometry in 16 patients showed decrease in both resting expiratory GEJ pressures and 4 s integrated pressures at 6 weeks after POEM compared to baseline. Furthermore, among POEM patients, there was a decrease in Eckardt scores from 7 (5–12) preoperatively to 1 (0–9; *P* < 0.01) at median 6 (1–18) months of follow-up. In conclusion, the authors demonstrated similar perioperative outcomes between POEM and LHM with excellent short-term results with the endoscopic approach.

Von Renteln and colleagues recently published the first international multicenter POEM study on 70 patients with achalasia from 5 different centers [22]. In their report, the mean length of the myotomy was 13 cm (range 5–23) and full thickness dissection into the mediastinum was observed in the majority of patients (69%). Treatment success, defined as Eckardt score of ≤3 at 3 months, was achieved in 97% of cases (95% CI 89–99), which persisted in 82.4% at 12 months. The authors reported a reduction in the mean LES pressure of 27.6 (24.2–31.0) to 8.9 (7.3–10.5) mmHg (*P* < 0.001) after POEM. This study demonstrates long-term symptomatic relief after POEM and confirms the efficacy of this technique as previously reported in single-center case series. 

### 3.2. POEM for the Treatment of Refractory Achalasia Patients in Setting of Prior Intervention

 Sharata et al. evaluated the safety and feasibility of POEM in 12 patients (9 achalasia) in the setting of prior endoscopic interventions (Botox injections, balloon dilations) [23]. POEM was successfully completed in all patients. The authors do report a single case of intramural bleeding requiring repeat endoscopy for hemostasis and another case of dehiscence at mucosotomy managed with endoscopic clipping. Overall, symptomatic relief was achieved in all patients based on a decrease in the median Eckardt score from 5 to 1 after POEM. When compared to 28 patients with no previous endoscopic intervention who underwent POEM, there were no differences in perioperative outcomes. The authors concluded that previous endoscopic therapies with Botox injections or large caliber balloon dilations do not increase adverse intraoperative or postoperative outcomes with POEM. 

Recurrence or persistence of symptoms occurs in approximately 10–20% of patients after Heller myotomy at 2 years of follow-up [11]. To date, there is no current consensus regarding optimal therapy for these patients. Sharata et al. [23] recently reported their experience on the efficacy and feasibility of POEM in patients after failed Heller myotomy. All 12 patients underwent successful POEM after a mean of 11.9 years (range 2–38 years) from the time of primary Heller myotomy. During a mean follow-up period of 10.4 months (range 5–14 months), treatment success was achieved in 11/12 patients (Eckardt score ≤3), with a reduction of pretreatment mean LES pressure of 29.4 mmHg to 13.5 mmHg following POEM (*P* < 0.001). There were no serious complications reported in the study, with only one case of small mucosal perforation at GEJ treated with endoclips, one case of pneumoperitoneum treated with needle decompression, and one patient requiring chest tube for symptomatic pneumothorax. In conclusion, the authors suggest that while endoscopic remyotomy is technically more challenging in this group of patients, POEM was still feasible and resulted in short-term symptom relief in >90% of the cases. 

### 3.3. Adverse Events Associated with POEM

POEM is a safe endoscopic technique associated with low rates of perioperative and postoperative complications. Dissection of air or CO_2_ into the mediastinum and/or peritoneum during submucosal tunneling and endoscopic myotomy has been commonly reported (Table 2). In most instances, pneumo-/capnoperitoneum can be followed clinically and when symptomatic, treated by decompression with Veress needle insertion [14, 15, 21]. There were 6 cases (out of a total of 94 patients) [14–21] of mucosal perforation at the gastroesophageal junction treated with endoscopic clipping without long-term adverse effects reported. Similarly, in their multicenter study of 70 patients, von Renteln et al. reported only one case of mucosal perforation at the mucosotomy site, which was managed successfully with endoscopic clipping. Perioperative bleeding is rare and endoscopic hemostasis is often successful. There have been some reports of delayed (several days following procedure) bleeding, which have been managed conservatively without requiring specific endoscopic or surgical intervention [20–22]. Cardiopulmonary complications are infrequent, although there was one case reported of aspiration pneumonia associated with prolonged postoperative recovery [17].

In contrast to these findings, in the largest published single-center POEM series to date, Zhou and colleagues [24] demonstrated a high incidence of POEM-specific complications. The authors analyzed the data of 119 patients with achalasia who underwent POEM from October 2010 to July 2011. Postoperative complications included pneumothorax (25.2%, 30/119), subcutaneous emphysema (55.5%, 66/119), mediastinal emphysema (29.4%, 35/119), pleural effusion (48.7%, 58/119), and pneumoperitoneum (39.5%, 47/119). The authors report that all post-POEM complications were managed effectively with traditional therapies without resorting to additional surgery. The discrepancy in the complication rate between this study and other series could potentially be attributed to differences on how the submucosal tunnel was created (posterior versus anterior esophageal wall) and the use of air versus CO_2_ for insufflation during POEM. 

Mucosotomy site closure can be technically challenging. Indeed, clip dislocation at the mucosal closure location requiring endoscopic reclipping was the most common complication reported by von Renteln in their multicenter study [22]. Recently, Saxena et al. [27] reported two cases in which closure of the mucosotomy could not be achieved with standard endoclips and proposed the use of over-the-scope clips (OTSC) for difficult cases. Similarly, it is our experience that OTSC may be an alternate method for closing the mucosal incision site when standard clipping has failed (Yang et al., Endoscopy 2013; ENDOS-2013-9120). Future studies evaluating the optimal approach and standardization of the technique in POEM are largely needed. 

### 3.4. Incidence of Gastroesophageal Reflux following POEM

 The reported short-term incidence of reflux symptoms following POEM is relatively low (range from 0% to 33%) [15–24]. This was primarily assessed based on patient self-reports of heartburn or reflux symptoms at follow-up, with one study using a validated questionnaire for GERD (Table 1). One study demonstrated high clinical reflux rates (37%) with 29% of patients still requiring maintenance therapy with proton-pump inhibitors [22]. In addition to clinical symptoms of reflux, Chiu et al. [17] demonstrated abnormal esophageal acid exposure on ambulatory pH monitoring in 20% (3/15 patients at 3-month follow-up). 

## 4. POEM versus Other Treatment Modalities for Achalasia

Initial data from these POEM studies highlight some potential advantages of this technique compared to endoscopic pneumatic balloon dilation. First, the short-term symptomatic efficacy of POEM is excellent. By following the principle of LHM, POEM provides a high lasting success rate (82% at 1 year) [22] and decreased need of retreatment when compared to balloon dilation. Second, the risk of uncontrolled perforation that may occur during balloon dilation could be avoided with myotomy under direct visualization. Based on the available evidence on POEM, it is evident that the longitudinal fibers are very thin and inadvertent transmural dissection can occur during endoscopic myotomy. However, “true” perforation is rare when mucosal integrity is maintained and closure of the mucosotomy site. Furthermore, the vast majority of reported cases of contained perforation with POEM have not required any specific intervention or have been managed successfully with endoscopic clipping. Thus, while POEM is associated with higher rates of pneumoperitoneum and subcutaneous emphysema perioperatively, there have been no reported significant clinical implications. Therefore, this initial clinical data on POEM seem to indicate that this is a procedure with an adequate risk profile; however, studies comparing the outcomes between POEM (with partial or complete myotomy) and endoscopic balloon dilation are needed before more definite conclusions can be drawn regarding long-term safety.

The potential advantages of POEM over LHM are founded on the basis of a myotomy through a natural orifice. First, advocates of POEM suggest that this technique allows the option of performing a very long myotomy. High mediastinal dissection can be challenging laparoscopically; thus, a transoral submucosal approach could presumably represent an alternate method for the management of conditions that may require extended myotomy, such as, vigorous achalasia. Unfortunately, current studies have been underpowered to detect differences in treatment response among different subtypes of achalasia. Furthermore, the operative techniques employed in most studies have been based on that described initially by Inoue and colleagues, and therefore they have not focused on the feasibility of creating a more extensive myotomy compared to LHM. Thus, whether a longer myotomy can be successfully performed and whether this will actually result in symptomatic improvement in different variant forms of achalasia remain to be elucidated. 

A second theoretical advantage of POEM over LMH is that the incisionless approach with the former technique should theoretically be associated with less operative risk and reduced postoperative pain. As previously described, Hungness et al. provided direct initial evidence that POEM has a safety profile comparable with LHM and is associated with shorter operative time and estimated blood loss. However, these improved parameters were minimal, and thus whether these differences translate into measureable clinical benefits is unclear. Interestingly, POEM was not associated with reduced postoperative pain compared to LHM. However, the caveats of this study include the nonrandomized and nonblinded nature of the study, which could have introduced bias or differences in baseline among the groups, accounting for some of the reported findings. 

Thirdly, some initial studies have suggested a potential role of POEM in the management of patients with refractory symptoms after surgical myotomy. Zhou and colleagues reported successful POEM in 12 patients with prior Heller myotomy and demonstrated excellent short-term symptom resolution and improvement in manometric outcomes. While this represents a very small sample size, it supports the potential application of POEM for patients who have failed previous open or laparoscopic myotomy. The role of POEM as an alternative treatment for persistent symptoms after Heller myotomy appears promising, especially in patients in which revisional operation may be complicated by periesophageal fibrotic scar tissue. Additional studies are needed to corroborate these positive initial findings.

Lastly, a transoral approach for myotomy potentially obviates the need to manipulate several structures around the GEJ in order to dissect the muscle layers. Studies have demonstrated a high incidence of GERD (up to 60% of patients) following LHM, and thus an adjuvant antireflux procedure is generally recommended [28]. This requirement adds a layer of complexity and intuitively increases the risk of complications. Endoscopic myotomy through the luminal side with POEM theoretically maintains the integrity of the natural antireflux mechanisms, which may account for an acceptable incidence (0 to 37%) of symptomatic GERD after POEM during short-term follow-up as described in most studies [15–24]. Future studies, using standardized validated clinical scores for GERD and objective assessment of esophageal acid exposure, are needed to investigate the long-term effects of POEM on acid reflux. 

## 5. Future Directions

Based on the initial clinical trials, POEM appears to be an effective therapy for achalasia with an acceptable safety profile. However, this procedure is both technically demanding and time consuming, even in the hands of experienced endoscopists [29]. Thus, further adaptations of this technique that may help accelerate the learning curve and reduce operative time are pivotal for promoting its applicability. 

Khashab and colleagues recently published their initial experience on a novel technique of “auto-tunneling” during POEM in a swine model [30]. In their swine model study, the authors used a submucosal lifting gel (Cook Medical, Winston-Salem, NC) as a dissecting gel, which was injected into the submucosal bleb created at the site of mucosotomy. Their results demonstrated that a complete submucosal tunnel to the level of the GEJ was noted to be already formed upon insertion of the endoscope into the submucosal space in all 5 pigs. Theoretically, this could obviate the need to mechanically dissect the submucosal loose areolar tissue in the esophagus during tunneling and thus significantly reduce the time and risk associated with “manual” tunneling. While future clinical studies are needed to validate these preliminary experimental findings, this study highlights the evolving nature of this field and introduces innovative modifications that may potentially circumvent current technical hurdles. 

 The tunneling approach in the submucosal space during POEM may be extended to other sites in the GI tract as well, extending the application of this technique to other areas such as endoscopic mucosal resection (EMR) and endoscopic submucosal dissection (ESD) for epithelial and subepithelial gastrointestinal lesions. Similarly, it can be envisioned that this technique may be used at other sphincter sites such as the pylorus and anorectum.

## 6. Conclusion

In summary, POEM appears to be a feasible endoscopic procedure for achalasia with excellent short-term symptom resolution and improvement in manometric outcomes. Initial studies have demonstrated that POEM can be conducted efficiently with a safety profile comparable to LHM, with the most commonly reported complication being uncomplicated pneumomediastinum and/or pneumoperitoneum. POEM is a promising, yet very sophisticated and technically demanding procedure that should be performed in centers with expertise and thoracic surgery support. Further prospective randomized trials are required to compare the effectiveness of POEM with current treatment modalities and to establish its long-term effects for the management of achalasia. 

## Figures and Tables

**Table 1:** Clinical trials evaluating POEM for the management of achalasia.

Authors	Study design	Patients (*n*)	Myotomy length; mean (range), cm	Dysphagia score		Eckardt score		LES pressure; mean (range), mm Hg		GERD symptoms	Follow-up; mean (range)
				Baseline; mean	Post-POEM; mean (*n*, %)	Baseline; mean	Post-POEM; mean (*n*, %)	Baseline	Post-POEM		
Inoue et al. [13]	Single-center prospective	17	8.1 (3–15)	10	1.3 (17, 100%)	N/A	N/A	52.4 (14.2–80.5)	19.8 (9.3–42.7)	N/A	5 (1–16) months
Swanström et al. [14]	Single-center prospective	5	10 (10–13)	N/A	0 or 1 (5, 100%)	N/A	N/A	36.5 (9.9– 39.8)	N/A	N/A	2 weeks
von Renteln et al. [15]	Single-center prospective	16	12 (8–17)	N/A	N/A	N/A	≤3 (15, 94%)	27.2	11.8	None	3 months
Costamagna et al. [16]	Single-center prospective	11	10.2 ± 2.8	N/A	N/A	7.1	1.1 (11, 100%)	45.1	16.9	None	3 months
Chiu et al. [17]	Single-center prospective	16	10.8	2 (range 0–2)	0 (range 0-1)	5.5 (range 0–8)	0 (range 0–3) (16, 100%)	43.6 ± 24.4	29.8 ± 22	1/16 (6.25%)	176.5 (98–230) days
Hungness et al. [21]	Single-center retrospective	18	9 (6–14)	N/A	N/A	7 (range 5–12)	1 (range 0–9) (18, 100%)	19 (7–51)	9 (0–23)	4/18 (22%) based on GerdQ score ≥7	6 (1–18) months
Zhou et al. [24]	Single-center prospective	12	10.1 (8–13)	N/A	N/A		≤3 (11, 91.7%)	29.4 (23.5–40.2)	13.5 (8.6–22.5)	1/12 (8.3%)	10.4 (5–14) months
Verlaan et al. [18]	Single-center prospective	10	N/A	N/A	N/A	8 (range 4–8)	1 (range 0-1) (10, 100%)	20.5 (15.8–24.3)	6.8 (4.0–12.0)	3/10 (30%)	3 months
Minami et al. [20]	Single-center retrospective	28	14.4 (10–18)	N/A	N/A	6.7 (range 2–12)	0.7 (range 0–3) (28; 100%)	71.2 (35.8–119.0)	21.0 (6.7–41.0)	6/28 (21.4%)	16 (3–28) months
Lee et al. [19]	Single-center prospective	13	8.5 (6–13)	N/A	N/A	6.4 (range 4–10)	0.4 (range 0–2) (13; 100%)	30.3 (14.4–57.1)	15.3 (9.6–22.3)	N/A	6.9 (1–14) months
Von Renteln et al. [22]	Multicenter prospective	70	13 (5–13)	N/A	N/A		≤3 (68, 97%) at 3 months	27.6 (24.2–31.0)	8.9 (7.3–10.5)	37%	10.1 (3–12) months

N/A: not available.

**Table 2:** Adverse events associated with POEM.

Authors	Patients (*n*)	Adverse events*, *n*	Additional treatment
Inoue et al. [13]	17	1 pneumoperitoneum	Veress needle

Swanström et al. [14]	5	2 small perforations in gastric cardia 3 capnoperitoneum	Endoscopic clips Veress needle

von Renteln et al. [15]	16	6 minor cutaneous emphysema 8 capnoperitoneum treated with Veress needle 1 small perforation at GEJ, treated with endoscopic clips	Veress needle Endoscopic clips

Costamagna et al. [16]	11	2 small perforations at junctional flap Asymptomatic pneumomediastinum in all patients 2 transient cervical emphysema	Endoscopic clips

Chiu et al. [17]	16	1 aspiration pneumonia 2 transient cervical emphysema	Hospital admission and monitoring

Hungness et al. [21]	18	7 capnoperitoneum 1 contained perforation at the EGJ	Veress needle

Ren et al. [25]	119	66 subcutaneous emphysema 30 pneumothorax 35 mediastinal emphysema 1 delayed hemorrhage 58 pleural effusions 59 inflammations/atelectases of the lungs 47 aeroperitonea	

Zhou et al. [24]	12	1 small perforation at GEJ, treated with endoscopic clips 3 capnoperitoneum, one treated with Veress needle 4 pneumothorax, one treated with thoracic tube 4 mediastinal emphysema 2 subcutaneous emphysema	Endoscopic clips Veress needle Thoracic tube

Verlaan et al. [18]	10	No complications reported	None

Minami et al. [20]	28	1 perforation at GEJ, treated with endoscopic clips 2 postoperative bleedings	Endoscopic clips

Lee et al. [19]	13	No complications reported	None

Von Renteln et al. [22]	70	3 clip dislocation at mucosal closure 1 perforation at mucosal entry site 3 mucosal injuries 1 bleeding requiring intervention 1 cap detached in submucosal tunnel 1 delayed bleeding (hematoma)	Endoscopic reclipping Endoscopic clips Endoscopic clips Endoscopic hemostasis Endoscopic removal Hospital admission and monitoring

*includes all reported outcomes, including nonclinically significant and asymptomatic adverse events.
